# Comparing Auditory and Visual Distractions for Reducing Pain Severity and Pain Anxiety in Older Outpatients with Burn: A Randomized Controlled Trial

**DOI:** 10.3390/geriatrics7030054

**Published:** 2022-04-30

**Authors:** Yaghob Moradipoor, Nahid Rejeh, Majideh Heravi Karimooi, Seyed Davood Tadrisi, Mostafa Dahmardehei, Tahereh Bahrami, Mojtaba Vaismoradi

**Affiliations:** 1 Faculty of Nursing and Midwifery, Shahed University, Tehran 1865133191, Iran; yaghobmoradipoor@gmail.com (Y.M.); heravi@shahed.ac.ir (M.H.K.); btahereh@rocketmail.com (T.B.); 2 Trauma Research Center, Faculty of Nursing, Baqiyatallah University of Medical Sciences, Tehran 1435916471, Iran; sdt1344@gmail.com; 3 Burn Research Center, Iran University of Medical Sciences, Tehran 1449614535, Iran; m.dahmardehei@gmail.com; 4 Faculty of Nursing and Health Sciences, Nord University, 8049 Bodø, Norway; mojtaba.vaismoradi@nord.no

**Keywords:** anxiety, burn, distraction, dressing change, older people, pain

## Abstract

Pain and anxiety are major issues among older patients with burn injuries. Complementary medicine and non-pharmacological methods can relieve pain and anxiety in older people, but comparison of the effects of these methods needs further research. This study aimed to compare the effects of auditory and visual distractions on pain severity and pain anxiety in older outpatients referred to a burn clinic for dressing change. In this randomized controlled clinical trial, older men were randomly assigned to three groups as auditory distraction, visual distraction, and control (*n* = 45 in each group). The Visual Analogue Scale (VAS) and the Burn Specific Pain Anxiety Scale (BSPAS) were used to asses pain severity and pain anxiety before and immediately after the interventions, and after wound dressing. Reduction in pain severity and pain anxiety after visual distraction was reported. Auditory distraction only reduced pain anxiety. Therefore, visual distraction had a better effect on alleviating pain anxiety compared with auditory distraction. Visual distraction is suggested to be used during dressing changes for older outpatients with burn injuries in outpatient clinics in order to reduce their burn-related suffering and improve their collaboration with the therapeutic regimen.

## 1. Introduction

Pain is a common complaint in patients with burn injuries [1]. Prolonged distress and painful procedures increase patients’ mortality, length of hospital stay, and related comorbidities including depression and posttraumatic stress disorder (PTSD) [1,2,3]. Age also influences the experience of pain in burn patients [1]. Older people are at high risk of burn injuries and related adverse outcomes due to cognitive impairment, low immunity, slow wound healing, and decreased mobility [4,5]. Moreover, painful diseases including osteoarthritis, diabetic neuropathy and lower back pain make older people more vulnerable to the negative consequences of burn injuries compared with younger adults [6,7].

Anxiety is a prevalent disorder in older patients, and they are at the high risk of anxiety disorders [8,9]. Changes in daily life routines and activities are the reasons that make older people more vulnerable to psychological problems including anxiety [10]. Pain anxiety is anxiety that patients feel before painful procedures including wound dressing [11]. Therefore, patients feel anxious because they predict that a painful intervention is going to happen [12]. Pain anxiety results in metabolic and immunologic disorders and interferes with the recovery process in patients with burn injuries [13].

The treatment of burn outcomes in older adults should be different from the treatment used for younger patients [14]. Poor management of pain and pain anxiety causes improper wound healing and also dissatisfaction with care among patients [13]. Opioid medications are the first treatment choice for the management of pain and anxiety in patients with burns [15,16]. However, they should be used with caution in older people because they are prone to significant adverse effects [17]. Complementary medicine and non-pharmacological treatments are safe therapeutic measures and can help with achieving treatment outcomes in patients with burns and reducing healthcare costs [18].

Distraction as a simple complementary and non-pharmacological approach can reduce pain severity and anxiety by drawing patients’ attention to pleasant stimuli [19,20,21]. Distraction inhibits the activity of the thalamus, insular cortex, and anterior cingulate cortex (ACC) and ultimately reduces pain [22]. Moreover, distraction improves patients’ feelings and helps them to feel more comfortable [23]. Older people can benefit from distraction techniques more than young adults, as they favor positive over negative stimuli [24]. Auditory distraction improves older adults’ cognitive impairment, physical activity, and overall wellbeing [25]. Visual distraction also provides a boost to older adults’ executive attention [26].

The effects of distraction techniques including virtual reality and music in burn patients have been investigated. For instance, a systematic review by Scapin et al. (2018) concluded that virtual reality using multisensory inputs such as visual, auditory, and tactile components had beneficial effects on patients with burns [27]. Furness et al. (2019) also suggested that virtual reality helped manage perceived pain in patients with burns [28]. Hsu et al. (2016) showed that music therapy was a valuable noninvasive intervention for the treatment of pain and pain anxiety during burn dressing [29]. Gezginci et al. (2018) also demonstrated that videos reduced pain severity and anxiety in patients with burns [30].

To the best of our knowledge, only a few studies have been conducted on the comparison of distraction techniques on reducing pain severity and pain anxiety among older adults with burns undergoing painful procedures. Therefore, this study sought to examine and compare the effects of auditory and visual distractions on pain severity and pain anxiety among older outpatients with burns.

## 2. Materials and Methods

### 2.1. Design and Subjects

This randomized controlled clinical trial with two parallel groups as intervention and control was carried out on older male patients. They were recruited from a burn hospital clinic where outpatients were referred for dressing changes from August 2019 to February 2020.

Inclusion criteria were: age 60–75 years; no cognitive impairment based on the Abbreviated Mental Test (AMT); no visual, verbal, and auditory impairment; no history of mental diseases; no history of burn injury; total burn surface area *<*25%; second-degree superficial burn; having burn injury only in limbs; being in the acute phase of burn injury within the first 72 h of incident; having no pain in other parts of the body; having pain score <3 according to the Visual Analogue Scale (VAS); no self-immolation; no drug abuse.

Painful experiences of previous burns and wound dressing affect pain severity and pain anxiety in adults with burns [31]. To avoid such a bias, we did not recruit participants with a previous history of burns. Additionally, those participants who used painkillers without prescription were excluded. Only older male patients were recruited, given the capacity to recruit the sample size according to the above-mentioned criteria and the need for homogeneity of the groups.

**Figure 1:**
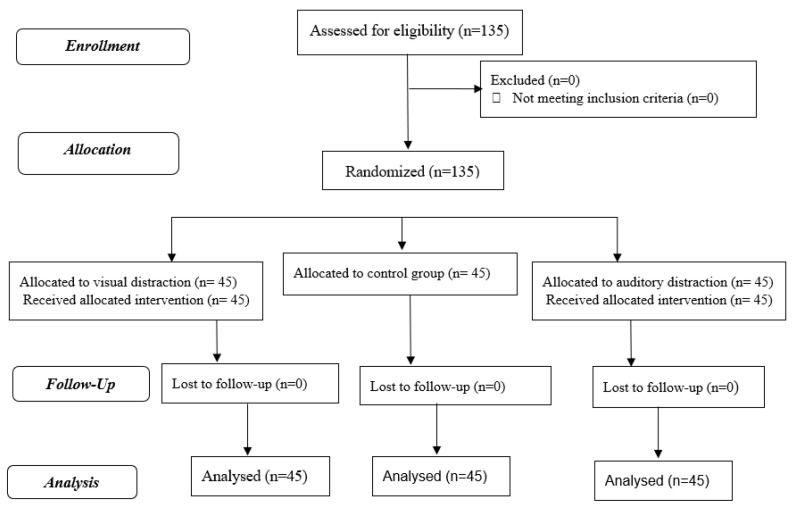
Process of the study according to the Consort flow diagram (2010).

The research protocol was registered on the Iranian Registry of Clinical Trials (decree code: IRCT20110912007529N21). The research has been reported based on the Consolidated Standards of Reporting Trials (CONSORT). The study process based on the CONSORT flow diagram is presented in Figure 1.

### 2.2. Sampling

A total of 135 older people were required using the power formula considering alpha of 0.05, power of 80%, and attrition rate of 15%.

Group = 3, total sample size before dropout = 39 × 3 = 117;

15% dropout t = 17.55 ≈ 18, total sample size after dropout = 117 + 18 = 135;

Total sample size per group 135/3 = 45.

After obtaining permission from the ethics committee affiliated with the university in which the second author (NR) worked, the first author (YM) assessed the patients’ medical records and recruited those participants who met the inclusion criteria. The older men were selected using a convenience sample method, and were randomly allocated to three groups as auditory distraction, visual distraction, and control using the block randomization method with the block size of 6 and with a 2:2:2 assignment ratio (see Supplementary Materials, File S1).

To conceal the allocation, the type of intervention was written on a piece of paper and placed in a sealed opaque envelope. In order to avoid bias, the statistical consultant of the study (SDT) generated the random allocation process. The envelopes were opened by the first author (YM) in the order of entry of the participants to the research to determine the group type.

There was no possibility of blinding the patients or researchers due to nature of the interventions. However, the data collector, who was one of the nursing staff working in the outpatient clinic, was blinded to the group assignments.

### 2.3. Interventions

#### 2.3.1. Auditory Distraction

Auditory distraction was started 20 min prior to dressing change [32]. The older patients listened to sounds from nature such as a flowing river, waterfall, walking through the forest, sea, and bird songs using headphones (Sony^®^ S820) and an MP3 player (Lenovo^®^) considering 25–50 dB sound volume calibrated by an audiologist, which was pretested and adjusted according to the participant’s comfort level. The auditory distraction continued until the end of the procedure.

#### 2.3.2. Visual Distraction

Initially 20 min before starting dressing change, natural and eye-catching images consisting of images of the sea, birds, and animals were broadcasted through a video display device on a laptop monitor in a manner that was easy for the participants to watch. The intervention continued until the end of dressing change.

#### 2.3.3. Control Group

Patients in this group received no intervention. It is noted that patients with burns referred to outpatient clinics are not prescribed any painkiller during therapeutic procedures.

### 2.4. Outcomes

The research outcomes were pain severity and pain anxiety reported by the participants in all three groups before and immediately after the interventions, and after wound dressing.

### 2.5. Data Collection

#### 2.5.1. Demographic Data Form

A demographic data collection questionnaire was developed. It consisted of questions about the participants’ age, marital status, occupation, medication use, percentage of burn, reason for burn, and the part of the body exposed to the burn injury.

#### 2.5.2. Abbreviated Mental Test (AMT)

Participants with cognitive disorders were identified using the AMT. It helped with the identification of any change in their cognitive function, and a score of 1 was given for each correct answer. Accordingly, a score of 0–3 suggested severe cognitive impairment, 4–7 moderate impairment, and ≥ 8 normal function [33]. The Cronbach’s alpha coefficient of the AMT for reliability checking was reported as 0.76 [34].

#### 2.5.3. Pain Severity

Pain severity was assessed using the VAS in all three groups. The VAS uses a 10 cm line, where extremely positive statements constitute one end and extremely negative statements are on the other end. The most positive and negative statements are scored 0 and 10, respectively. Scores of 1–3 represent low pain, 4–6 medium pain, and 7–9 severe pain. Hoggart and Williamson (2005) revealed that this scale was a reliable and valid pain rating scale that could be used in clinical practice [35].

#### 2.5.4. Burn Specific Pain Anxiety Scale (BSPAS)

Pain anxiety was assessed using the BSPAS in all three groups. It is a self-reporting scale that has been developed by Tall and Faber (1999) to analyze anxiety associated with the anticipation of pain during or after various medical procedures, including dressing change. The participants were asked to answer the nine-item BSPAS and show their responses to each item on a 0–100 visual analogue scale ranging from "not at all" to "the worst imaginable way". The total BSPAS score was calculated as the mean of the nine items scored on the VAS, and a mean score >50 indicated the highest anxiety. This scale has high internal reliability (α = 0.90) [36]. Moreover, Najafi et al. (2013) translated and evaluated the reliability and validity of the Farsi version of the BSPAS and reported a satisfactory Cronbach’s α coefficient of 0.96 [37].

### 2.6. Statistical Data Analysis

SPSS software v.21.0 was used to analyze the collected data. The sociodemographic and burn-related characteristics of the participants were given as the number and percentage of distributions. To examine pain severity and pain anxiety, a normal distribution conformity analysis via the Kolmogorov-Smirnov test was performed. A generalized estimating equation was used to examine the effects of distractions on pain severity. Repeated measure ANOVA was used to compare the effects of the interventions on pain anxiety in different time intervals. Cohen’s d test was also performed to analyze the effect of each distraction technique on alleviating pain anxiety. The statistical significance level was *p* < 0.05.

### 2.7. Ethical Considerations

The research was approved by the ethics committee of Shahed University (decree code: IR.SHAHED.REC.1398.049). The aims and methods of the study were explained to the participants, who provided verbal and written informed consent. Confidentiality and anonymity of the participants were ensured by using numbers instead of names. Additionally, they were informed that they could withdraw from the study without any impact on their care process in the outpatient clinic.

## 3. Results

All of the 135 eligible participants that were allocated to the groups completed the research.

### 3.1. Demographic Characteristics of the Participants

The participants’ mean age was 67.01 ± 4.82 years, and their mean percentage of burn was 62.86% ± 8.36%. There were no statistically significant differences between the groups in terms of patient characteristics (*p* > 0.05), indicating homogeneity of the groups (Table 1).

### 3.2. Pain Severity

The mean scores of pain severity in the groups are shown in Table 2. Pain severity was decreased in the visual distraction group after the intervention (6.06 ± 0.91), which was continued after dressing change (6.62 ± 1.19). No change in pain severity was observed after auditory distraction.

### 3.3. Pain Anxiety

Auditory and visual distractions alleviated pain anxiety in the groups (*p* < 0.05) (Table 3). However, visual distraction had a larger effect (*d* = 1) on pain anxiety than auditory distraction, which had a medium effect (*d* = 0.6).

## 4. Discussion

Successful pain management in older patients with burn injuries requires a comprehensive and safe care approach that avoids the possible side effect of painkillers [15]. Distraction techniques have the potential to alleviate the symptoms of burns including pain and pain anxiety [38,39]. The results of this experimental study demonstrated that auditory distraction did not have a significant effect on pain severity, but decreased pain anxiety among older patients with burns. However, visual distraction reduced pain anxiety more than auditory distraction after the intervention and also after wound dressing.

Similarly to the results of the current study, a clinical trial on children compared the effects of visual and auditory distractions on physiological indicators and pain intensity. It concluded that visual distraction reduced pain intensity more than auditory distraction [40]. Moreover, a randomized controlled trial compared three distraction therapies including video, music, and stress balls during cystoscopy. Pain intensity, anxiety, and satisfaction scores during the procedure were significantly lower in the video group compared with the music and stress ball groups [30]. Another clinical trial reported that visual distraction improved satisfaction and decreased anxiety and pain in patients undergoing colonoscopy [41].

The results of this clinical trial demonstrated that visual distraction alleviated pain severity and pain anxiety. Oliveira et al. (2016) reported that audiovisual distraction effectively reduced the intensity of pain perception in pediatric inpatients [42]. Lue et al. (2018) performed a systematic review and meta-analysis and reported that virtual reality was an effective pain reduction measure for burn patients undergoing dressing change [43]. A randomized controlled trial by Alhlib et al. (2020) revealed that patients watching video clips with music during local rigid cystoscopy experienced a lower level of pain than the control group [44].

In this research, auditory distraction did not reduce pain intensity. Nevertheless, auditory distraction had a medium effect on pain anxiety. A systematic review and meta-analysis of randomized controlled trials in burn patients on the effect of music during treatment showed a positive correlation between music interventions and pain alleviation, anxiety relief, and heart rate reduction in burn patients [39]. Moreover, different studies have reported the positive effect of music on reducing pain and anxiety in burn patients [29,45,46]. A clinical trial on the efficacy of music-based imagery and musical debridement showed a significant reduction in the self-reporting of pain in those patients who received music therapy. However, their anxiety was not significantly relieved [47]. Another study was undertaken to investigate the effect of relaxation music on the perceived levels of pain and anxiety during a range of motion exercises, but it did not indicate any statistically significant reduction in anxiety or pain during the exercises [48]. A study concerning the comparison of sensory focusing, music distraction, and usual care during dressing change in burn patients indicated that assessing pain after a delay might improve the efficacy of music distraction [49]. Therefore, a probable reason for the ineffectiveness of auditory distraction in our clinical trial could have been the immediate assessment of pain severity. Moreover, wider musical selections could mitigate pain intensity in the participants. Another possible explanation for these controversies may be the participants’ age. Older adults usually suffer from chronic pain [50] that can worsen the perceived pain of burn treatments. The contradictions of these studies highlight the importance of repeating trials concerned with the effect of distraction techniques in older patients with burns.

A limitation of this research was that it was only performed on older male patients to achieve homogeneity in the study groups. Therefore, our research results cannot be generalized to older female patients. As gender is an important factor in the modulation of pain and anxiety [51,52], it is highly recommended to repeat future trials on older female patients with burn injuries. The patient’s perception of pain is influenced by their character and cultural and emotional status, which might have impacted our research results. Moreover, pain and anxiety measurements in this study were subjective and relied on self-reporting. However, this study was performed in a burn hospital clinic where outpatients were referred for dressing change, and nurses and other caregivers did not have the chance to assess pain severity objectively in older adults with burns. Objective methods such as monitoring older adults’ vital signs and neurophysiological parameters are suggested in future studies to measure patients’ feelings more precisely during dressing change.

## 5. Conclusions

There was a statistically significant reduction in pain severity and pain anxiety after visual distraction among older outpatients with burn injuries. However, the auditory distraction only reduced pain anxiety. Visual distraction is suggested to be used during dressing change for older outpatients with burns in outpatient clinics in order to reduce their burn-related suffering and improve their collaboration with the therapeutic regimen.

As few studies can be found in the international literature on burn outcomes in older adults, further research is needed to assess and compare the effects of visual and auditory distraction techniques on physical and psychological indicators among older adults with burn injuries.

## Figures and Tables

**Table 1:** Comparison of the demographic characteristics of the participants between the groups (*n* = 135).

Variables	Groups			Test, *p*-Value
	Auditory Distraction Mean ± SD	Visual Distraction Mean ± SD	Control Mean ± SD	
Age, year	66.07 ± 5.24	67.69 ± 4.22	67.56 ± 5.02	H (2) = 3.36
				*p* * = 0.18
Percentage of the burn, %	76.79 ± 8.4	49.34 ± 8.4	62.45 ± 8.3	H (2) = 0.52
				*p* = 0.77
**Characteristic**	**Auditory Distraction** ***n*** **(%)**	**Visual Distraction** ***n*** **(%)**	**Control** ***n*** **(%)**	**Test, *p*-value**
Marital Status				
Married	33 (24.4)	32 (23.7)	38 (28.1)	X^2^(2) = 2.53 *p* ** = 0.28
Widower/divorced	12 (8.9)	13 (9.6)	7 (5.2)	
Education Level				
Illiterate	2 (1.5)	11 (8.1)	7 (5.2)	X^2^(6) = 11.21 *p* = 0.08
Elementary	25 (18.5)	16 (11.9)	17 (12.6)	
Diploma	15 (11.1)	11 (8.1)	16 (11.9)	
Academic	3 (2.2)	7 (5.2)	5 (3.7)	
Occupation				
Employee	6 (4.4)	13 (9.6)	4 (3)	X^2^(4) = 9.29 *p* = 0.05
Self-employment	18 (13.3)	9 (6.7)	16 (11.9)	
Retired	21 (15.6)	23 (17)	25 (18.5)	
Living Condition				
Alone	13 (9.6)	9 (6.7)	16 (11.9)	X^2^(4) = 8.34 *p* = 0.08
With spouse	21 (15.6)	23 (17)	25 (18.5)	
With spouse and children	11 (8.1)	19 (14.1)	16 (11.9)	
Smoking				
Yes	26 (19.3)	22 (16.3)	32 (23.7)	X^2^(2) = 4.66 *p* = 0.09
No	19 (14.1)	23 (17)	13 (9.6)	
Reason for the Burn				
Hot object, chemical material, electricity	16 (11.9)	17 (12.6)	16 (11.9)	X^2^(4) = 0.34 *p* = 0.98
Hot liquid	15 (11.1)	15 (11.1)	17 (12.6)	
Area of the Burn				
Having single burn (hand or foot)	12 (8.9)	20 (14.8)	12(8.9)	X^2^(2) = 4.31 *p* = 0.11
Having multiple burns (hand and foot)	33 (24.4)	25 (18.5)	33(24.4)	
Painkiller Use				
Acetaminophen or Gelofen	21 (15.6)	23 (17)	24 (17.8)	X^2^(2) = 0.41 *p* = 0.81
No medication	24 (17.8)	22 (16.6)	21 (15.6)	

* Kruskal-Wallis; ** Chi-square.

**Table 2:** Comparison of the mean scores of pain severity between the groups (*n* = 135).

Groups.	Before the Intervention Mean ± SD	After the Intervention Mean ± SD	After Wound Dressing Mean ± SD
Auditory Distraction	7.77 ± 0.70	7.08 ± 1.12	7.17 ± 1.00
	Severe pain	Severe pain	Severe pain
Visual Distraction	7.75 ± 0.64	6.06 ± 0.91	6.62 ± 1.19
	Severe pain	Moderate pain	Moderate pain
Control	7.60 ± 0.86	7.73 ± 0.75	7.68 ± 0.87
	Severe pain	Severe pain	Severe pain
*p*-value ^a^	**Group**	**Time**	**Group × time**
	W ^b^ (2) = 79.85 *p* = 0.001	W (1) =2.96 *p* = 0.08	W (2) =7.54 *p* = 0.02

^a^ Generalized estimating equation, ^b^ Wald chi-square.

**Table 3:** Comparison of the mean scores of pain anxiety between the groups (*n* = 135).

Study Group	Before the Intervention	After the Intervention	After Wound Dressing	Statistical Test	Effect Size
Auditory distraction	70.22 ± 6.49	64.37 ± 9.59	66.91 ± 6.11	M ^a^ (2) = 0.80	ή2 ^c^ = 0.09
				*p* = 0.009	
				F ^b^ (1.6, 73.5) = 7.48	d ^d^ = 0.6
				*p* = 0.002	
Visual distraction	70.20 ± 5.64	62.55 ± 6.98	65.11 ± 6.46	M (2) = 0.97	ή2 = 0.20
				*p* = 0.62	
				F (2, 88) = 17.72	d = 1
				*p* = 0.001	
Control	70.86 ± 6.87	70.51 ± 7.65	70.33 ± 7.52	M (2) = 0.98	
				*p* = 0.68	
				F (2, 88) = 0.08	
				*p* = 0.91	
	**Group**			**Group × time**	
	F (2, 264) = 17.23, *p* = 0.001			F (4, 264) = 3.88	ή2 = 0.063, d = 0.51
				*p* = 0.001	
	M (2) = 0.97, *p* = 0.62				

^a^ Mauchly’s sphericity; ^b^ Repeated measures ANOVA; ^c^ Eta-squared; ^d^ Cohen’s d.

## Data Availability

All data related to this research have been presented in this article.

## Supplementary Materials

The following supporting information can be downloaded at: https://www.mdpi.com/article/10.3390/geriatrics7030054/s1, File S1: Block randomization method.

## References

[1] Mengistu N.D., Obsa M.S., Gemeda L.A. (2018). Burn Pain Management at Burn Unit of Yekatit 12 Hospitals, Addis Ababa. Pain Res. Treat..

[2] Myers R., Lozenski J., Wyatt M., Peña M., Northrop K., Bhavsar D., Kovac A. (2017). Sedation and Analgesia for Dressing Change: A Survey of American Burn Association Burn Centers. J. Burn Care Res.: Off. Publ. Am. Burn Assoc..

[3] Spronk I., Legemate C., Oen I., van Loey N., Polinder S., van Baar M. (2018). Health related quality of life in adults after burn injuries: A systematic review. PLoS ONE.

[4] Lundgren R.S., Kramer C.B., Rivara F.P., Wang J., Heimbach D.M., Gibran N.S., Klein M.B. (2009). Influence of comorbidities and age on outcome following burn injury in older adults. J. Burn Care Res..

[5] Rowan M.P., Cancio L.C., Elster E.A., Burmeister D.M., Rose L.F., Natesan S., Chan R.K., Christy R.J., Chung K.K. (2015). Burn wound healing and treatment: Review and advancements. Crit Care.

[6] Goei H., van Baar M.E., Dokter J., Vloemans J., Beerthuizen G.I.J.M., Middelkoop E., van der Vlies K.H. (2020). Burns in the elderly: A nationwide study on management and clinical outcomes. Burn. Trauma.

[7] Jeschke M.G., Peck M.D. (2017). Burn Care of the Elderly. J. Burn Care Res.: Off. Publ. Am. Burn Assoc..

[8] Bryant C., Jackson H., Ames D. (2008). The prevalence of anxiety in older adults: Methodological issues and a review of the literature. J. Affect. Disord..

[9] Magnon V., Dutheil F., Vallet G.T. (2021). Benefits from one session of deep and slow breathing on vagal tone and anxiety in young and older adults. Sci. Rep..

[10] Kazeminia M., Salari N., Vaisi-Raygani A., Jalali R., Abdi A., Mohammadi M., Daneshkhah A., Hosseinian-Far M., Shohaimi S. (2020). The effect of exercise on anxiety in the elderly worldwide: A systematic review and meta-analysis. Health Qual. Life Outcomes.

[11] Fallah L.Y., Ahmadi A., Ruche A.B., Taremiha A., Soltani N., Mafi M. (2019). The effect of early change of skin graft dressing on pain and anxiety among burn patients: A two-group randomized controlled clinical trial. Int. J. Burn. Trauma.

[12] Wang Y., Wu B., Ma S., Wang D., Gan T., Liu H., Yang Z. (2022). Effect of mapping characteristic on audiovisual warning: Evidence from a simulated driving study. Appl. Ergon..

[13] Najafi Ghezeljeh T., Mohades Ardebili F., Rafii F. (2017). The effects of massage and music on pain, anxiety and relaxation in burn patients: Randomized controlled clinical trial. Burn. J. Int. Soc. Burn Inj..

[14] Pham T.N., Kramer C.B., Wang J., Rivara F.P., Heimbach D.M., Gibran N.S., Klein M.B. (2009). Epidemiology and outcomes of older adults with burn injury: An analysis of the National Burn Repository. J. Burn Care Res.: Off. Publ. Am. Burn Assoc..

[15] James D.L., Jowza M. (2017). Principles of Burn Pain Management. Clin. Plast. Surg..

[16] Rohilla L., Agnihotri M., Trehan S.K., Sharma R.K., Ghai S. (2018). Effect of Music Therapy on Pain Perception, Anxiety, and Opioid Use During Dressing Change Among Patients With Burns in India: A Quasi-experimental, Cross-over Pilot Study. Ostomy/Wound Manag..

[17] Abdulla A., Adams N., Bone M., Elliott A.M., Gaffin J., Jones D., Knaggs R., Martin D., Sampson L., Schofield P. (2013). Guidance on the management of pain in older people. Age Ageing.

[18] De Silva A.P., Niriella M.A., Nandamuni Y., Nanayakkara S.D., Perera K.R.P., Kodisinghe S.K., Subasinghe K.C.E., Pathmeswaran A., de Silva H.J. (2016). Effect of audio and visual distraction on patients undergoing colonoscopy: A randomized controlled study. Endosc. Int. Open.

[19] Aghbolagh M.G., Bahrami T., Rejeh N., Heravi-Karimooi M., Tadrisi S.D., Vaismoradi M. (2020). Comparison of the Effects of Visual and Auditory Distractions on Fistula Cannulation Pain among Older Patients Undergoing Hemodialysis: A Randomized Controlled Clinical Trial. Geriatrics.

[20] Azarmnejad E., Sarhangi F., Javadi M., Rejeh N., Amirsalari S., Tadrisi S.D. (2017). The effectiveness of familiar auditory stimulus on hospitalized neonates’ physiologic responses to procedural pain. Int. J. Nurs. Pract..

[21] Xiaolian J., Xiaolin L., Lan Z.H. (2015). Effects of visual and audiovisual distraction on pain and anxiety among patients undergoing colonoscopy. Gastroenterol. Nurs.: Off. J. Soc. Gastroenterol. Nurses Assoc..

[22] Johnson M.H. (2005). How does distraction work in the management of pain?. Curr. Pain Headache Rep..

[23] Hu W., Yang K., Zhang L., Lu X. (2021). Effect of media distraction (audio-visual and music) for pain and anxiety control in patients undergoing shock-wave lithotripsy: A systematic review and meta-analysis. Exp. Ther. Med..

[24] Martins B., Sheppes G., Gross J.J., Mather M. (2018). Age Differences in Emotion Regulation Choice: Older Adults Use Distraction Less Than Younger Adults in High-Intensity Positive Contexts. J. Gerontology. Ser. Bpsychological Sci. Soc. Sci..

[25] Clark I.N., Baker F.A., Taylor N.F. (2016). Older Adults’ Music Listening Preferences to Support Physical Activity Following Cardiac Rehabilitation. J. Music Ther..

[26] Gamble K.R., Howard J.H.J., Howard D.V. (2014). Not just scenery: Viewing nature pictures improves executive attention in older adults. Exp. Aging Res..

[27] Scapin S., Echevarría-Guanilo M.E., Boeira Fuculo Junior P.R., Gonçalves N., Rocha P.K., Coimbra R. (2018). Virtual Reality in the treatment of burn patients: A systematic review. Burn.: J. Int. Soc. Burn Inj..

[28] Furness P.J., Phelan I., Babiker N.T., Fehily O., Lindley S.A., Thompson A.R. (2019). Reducing Pain During Wound Dressings in Burn Care Using Virtual Reality: A Study of Perceived Impact and Usability With Patients and Nurses. J. Burn Care Res.: Off. Publ. Am. Burn Assoc..

[29] Hsu K.-C., Chen L.F., Hsiep P.H. (2016). Effect of music intervention on burn patients’ pain and anxiety during dressing changes. Burn.: J. Int. Soc. Burn Inj..

[30] Gezginci E., Iyigun E., Kibar Y., Bedir S. (2018). Three Distraction Methods for Pain Reduction During Cystoscopy: A Randomized Controlled Trial Evaluating the Effects on Pain, Anxiety, and Satisfaction. J. Endourol..

[31] Byers J.F., Bridges S., Kijek J., LaBorde P. (2001). Burn patients’ pain and anxiety experiences. J. Burn Care Rehabil.

[32] Mohammadi Fakhar F., Rafii F., Jamshidi Orak R. (2013). The effect of jaw relaxation on pain anxiety during burn dressings: Randomised clinical trial. Burn.: J. Int. Soc. Burn Inj..

[33] Faraji J., Khoshknab M.F., Khankeh H. (2013). The effect of poetry therapy on the cognitive status in elderly residents of a nursing home. Complementary Med. J. Fac. Nurs. Midwifery.

[34] Bakhtiyari F., Foroughan M., Fakhrzadeh H., Nazari N., Najafi B., Alizadeh M., Arzaghi M., Sharifi F., Shoaee S., Mostafa Q. (2014). Validation of the persian version of Abbreviated Mental Test (AMT) in elderly residents of Kahrizak charity foundation. Iran. J. Diabetes Metab..

[35] Williamson A., Hoggart B. (2005). Pain: A review of three commonly used pain rating scales. J. Clin. Nurs..

[36] Taal L.A., Faber A.W., van Loey N.E., Reynders C.L., Hofland H.W. (1999). The abbreviated burn specific pain anxiety scale: A multicenter study. Burn.: J. Int. Soc. Burn Inj..

[37] Ghezeljeh T.N., Ardebili F.M., Rafii F., Hagani H. (2013). Translation and psychometric evaluation of Persian versions of Burn Specific Pain Anxiety Scale and Impact of Event Scale. Burn.: J. Int. Soc. Burn Inj..

[38] Chu H., Brailey R., Clarke E., Sen S.K. (2021). Reducing pain through distraction therapy in small acute paediatric burns. Burns.

[39] Li J., Zhou L., Wang Y. (2017). The effects of music intervention on burn patients during treatment procedures: A systematic review and meta-analysis of randomized controlled trials. Bmc Complementary Altern. Med..

[40] Cheraghi F., Kalili A., Soltanian A., Eskandarlou M., Sharifian P. (2021). A Comparison of the Effect of Visual and Auditory Distractions on Physiological Indicators and Pain of Burn Dressing Change Among 6-12-Year-Oldchildren: A Clinical Trial Study. J. Pediatric Nurs..

[41] Umezawa S., Higurashi T., Uchiyama S., Sakai E., Ohkubo H., Endo H., Nonaka T., Nakajima A. (2015). Visual distraction alone for the improvement of colonoscopy-related pain and satisfaction. World J. Gastroenterol.

[42] Oliveira N.C.A.C., Santos J.L.F., Linhares M.B.M. (2017). Audiovisual distraction for pain relief in paediatric inpatients: A crossover study. Eur. J. Pain (Lond. Engl.).

[43] Luo H., Cao C., Zhong J., Chen J., Cen Y. (2019). Adjunctive virtual reality for procedural pain management of burn patients during dressing change or physical therapy: A systematic review and meta-analysis of randomized controlled trials. Wound Repair Regen. Off. Publ. Wound Heal. Soc. Eur. Tissue Repair Soc..

[44] Alhlib A.R., Haffejee M., Nel M.J. (2021). Pain modulation by audiovisual distraction during cystoscopy. Urol. Ann..

[45] Najafi Ghezeljeh T., Mohades Ardebili F., Rafii F., Manafi F. (2017). The Effect of Massage on Anticipatory Anxiety and Procedural Pain in Patients with Burn Injury. World J. Plast. Surg..

[46] Tan X., Yowler C.J., Super D.M., Fratianne R.B. (2010). The efficacy of music therapy protocols for decreasing pain, anxiety, and muscle tension levels during burn dressing changes: A prospective randomized crossover trial. J. Burn Care Res.: Off. Publ. Am. Burn Assoc..

[47] Fratianne R.B., Prensner J.D., Huston M.J., Super D.M., Yowler C.J., Standley J.M. (2001). The effect of music-based imagery and musical alternate engagement on the burn debridement process. J. Burn Care Rehabil..

[48] Ferguson S.L., Voll K.V. (2004). Burn pain and anxiety: The use of music relaxation during rehabilitation. J. Burn Care Rehabil..

[49] Haythronthwaite J.A., Lawrence J.W., Fauerbach J.A. (2001). Brief cognitive interventions for burn pain. Ann. Behav. Med.: A Publ. Soc. Behav. Med..

[50] Schwan J., Sclafani J., Tawfik V.L. (2019). Chronic Pain Management in the Elderly. Anesth. Clin.

[51] Fernie B.A., Wright T., Caselli G., Nikčević A.V., Spada M.M. (2017). Metacognitions as Mediators of Gender Identity-related Anxiety. Clin. Psychol. Psychother..

[52] Pieretti S., Di Giannuario A., Di Giovannandrea R., Marzoli F., Piccaro G., Minosi P., Aloisi A.M. (2016). Gender differences in pain and its relief. Ann. Dell’istituto Super. Di Sanita.

