# Supplementary material for: Comparing Auditory and Visual Distractions for Reducing Pain Severity and Pain Anxiety in Older Outpatients with Burn: A Randomized Controlled Trial
Source: Geriatrics (Basel). 2022 Apr 30;7(3):54. doi: 10.3390/geriatrics7030054 (PMC9149902; doi:10.3390/geriatrics7030054)
Supplement: Supplementary file 1 [file geriatrics-07-00054-s001.zip › geriatrics-1673017-supplementary.pdf]

Seed: 27036868549660

Block sizes: 6

Actual list length: 138

block identifier, block size, sequence within block, treatment

- 1, 6, 1, Group C
- 1, 6, 2, Group A
- 1, 6, 3, Group C
- 1, 6, 4, Group B
- 1, 6, 5, Group A
- 1, 6, 6, Group B
- 2, 6, 1, Group B
- 2, 6, 2, Group A
- 2, 6, 3, Group C
- 2, 6, 4, Group C
- 2, 6, 5, Group A
- 2, 6, 6, Group B
- 3, 6, 1, Group A
- 3, 6, 2, Group B
- 3, 6, 3, Group B
- 3, 6, 4, Group C
- 3, 6, 5, Group C
- 3, 6, 6, Group A
- 4, 6, 1, Group B
- 4, 6, 2, Group C
- 4, 6, 3, Group B
- 4, 6, 4, Group C
- 4, 6, 5, Group A
- 4, 6, 6, Group A
- 5, 6, 1, Group A
- 5, 6, 2, Group B
- 5, 6, 3, Group A
- 5, 6, 4, Group C
- 5, 6, 5, Group C
- 5, 6, 6, Group B
- 6, 6, 1, Group B
- 6, 6, 2, Group B
- 6, 6, 3, Group C
- 6, 6, 4, Group A

- 6, 6, 5, Group C
- 6, 6, 6, Group A
- 7, 6, 1, Group C
- 7, 6, 2, Group B
- 7, 6, 3, Group A
- 7, 6, 4, Group B
- 7, 6, 5, Group A
- 7, 6, 6, Group C
- 8, 6, 1, Group A
- 8, 6, 2, Group A
- 8, 6, 3, Group C
- 8, 6, 4, Group B
- 8, 6, 5, Group B
- 8, 6, 6, Group C
- 9, 6, 1, Group B
- 9, 6, 2, Group B
- 9, 6, 3, Group A
- 9, 6, 4, Group C
- 9, 6, 5, Group C
- 9, 6, 6, Group A
- 10, 6, 1, Group C
- 10, 6, 2, Group A
- 10, 6, 3, Group A
- 10, 6, 4, Group B
- 10, 6, 5, Group B
- 10, 6, 6, Group C
- 11, 6, 1, Group B
- 11, 6, 2, Group C
- 11, 6, 3, Group A
- 11, 6, 4, Group C
- 11, 6, 5, Group B
- 11, 6, 6, Group A
- 12, 6, 1, Group B
- 12, 6, 2, Group A
- 12, 6, 3, Group C
- 12, 6, 4, Group A
- 12, 6, 5, Group B
- 12, 6, 6, Group C
- 13, 6, 1, Group B
- 13, 6, 2, Group B
- 13, 6, 3, Group A

- 13, 6, 4, Group C
- 13, 6, 5, Group C
- 13, 6, 6, Group A
- 14, 6, 1, Group B
- 14, 6, 2, Group A
- 14, 6, 3, Group C
- 14, 6, 4, Group A
- 14, 6, 5, Group B
- 14, 6, 6, Group C
- 15, 6, 1, Group C
- 15, 6, 2, Group A
- 15, 6, 3, Group B
- 15, 6, 4, Group C
- 15, 6, 5, Group B
- 15, 6, 6, Group A
- 16, 6, 1, Group C
- 16, 6, 2, Group B
- 16, 6, 3, Group A
- 16, 6, 4, Group C
- 16, 6, 5, Group B
- 16, 6, 6, Group A
- 17, 6, 1, Group B
- 17, 6, 2, Group C
- 17, 6, 3, Group A
- 17, 6, 4, Group C
- 17, 6, 5, Group B
- 17, 6, 6, Group A
- 18, 6, 1, Group B
- 18, 6, 2, Group A
- 18, 6, 3, Group C
- 18, 6, 4, Group B
- 18, 6, 5, Group A
- 18, 6, 6, Group C
- 19, 6, 1, Group B
- 19, 6, 2, Group C
- 19, 6, 3, Group A
- 19, 6, 4, Group A
- 19, 6, 5, Group B
- 19, 6, 6, Group C
- 20, 6, 1, Group B
- 20, 6, 2, Group A

- 20, 6, 3, Group C
- 20, 6, 4, Group C
- 20, 6, 5, Group A
- 20, 6, 6, Group B
- 21, 6, 1, Group B
- 21, 6, 2, Group A
- 21, 6, 3, Group C
- 21, 6, 4, Group B
- 21, 6, 5, Group C
- 21, 6, 6, Group A
- 22, 6, 1, Group A
- 22, 6, 2, Group B
- 22, 6, 3, Group A
- 22, 6, 4, Group B
- 22, 6, 5, Group C
- 22, 6, 6, Group C
- 23, 6, 1, Group C
- 23, 6, 2, Group B
- 23, 6, 3, Group C
